# Environmental chemicals and the tumor immune microenvironment

**DOI:** 10.1002/imt2.70160

**Published:** 2026-07-22

**Authors:** Tongtong Zhang, Yanxia Hu, Weiran Zhang, Yang Jin, Chaoyang Sun, Afshin Samali, Shunqing Xu, Xia Sheng

**Affiliations:** ^1^ Institute of Biomedical Research, School of Life and Health Sciences Hainan University Haikou China; ^2^ School of Basic Medical Sciences Chongqing Medical University Chongqing China; ^3^ Department of Biosciences University of Oslo Oslo Norway; ^4^ Cancer Biology Research Center (Key Laboratory of the Ministry of Education), Tongji Hospital, Tongji Medical College Huazhong University of Science and Technology Wuhan China; ^5^ Apoptosis Research Centre, School of Biological and Chemical Sciences University of Galway Galway Ireland; ^6^ Department of Environmental Health, School of Environmental Science and Technology Hainan University Haikou China

## Abstract

Impact of representative environmental chemicals on tumor immune microenvironment (TIME). Representative environmental chemicals are ubiquitously distributed across air, soil, aquatic systems, and biota. These toxicants enter the human body via multiple exposure routes, including inhalation, dietary intake, and dermal absorption, subsequently accumulating in vital organs (such as the liver, lungs, kidneys, and intestines) as well as within tumor tissues. By disrupting physiological homeostasis, they impair the functionality of critical biological systems, including the metabolic, nervous, circulatory, and immune systems. Notably, within tumor tissues, these chemicals remodel the TIME by suppressing antitumor immunity and compromising the efficacy of immunotherapy, thereby promoting tumor initiation and progression.

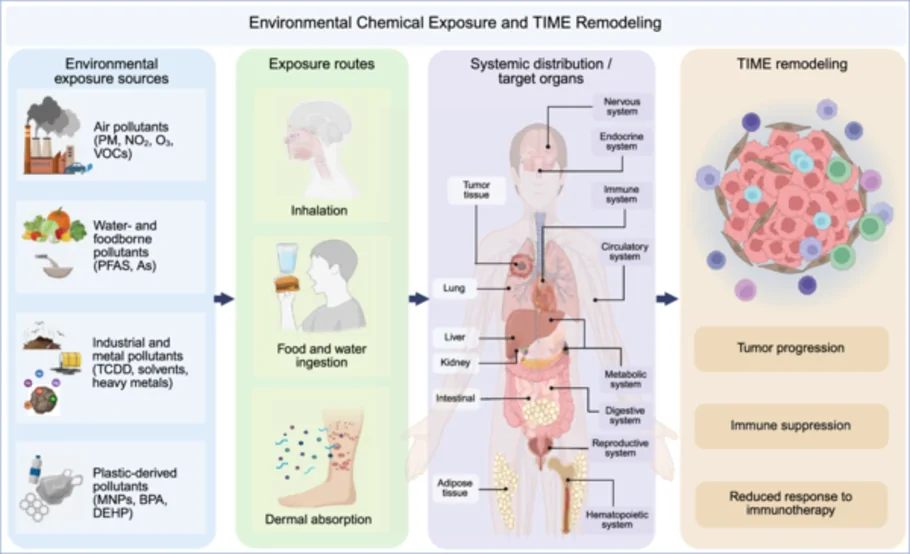

The tumor immune microenvironment (TIME) is shaped by a complex interplay of (epi)genetic and environmental factors, and plays a central role in the initiation and progression of human cancers [1]. While intrinsic regulators of the TIME have been extensively studied, the contribution of extrinsic factors, particularly environmental chemicals, has received far less attention. Environmental chemicals are widely distributed, subjecting humans to frequent or chronic exposure with insidious health consequences. Increasing evidence indicates that such chemicals are not only mutagenic and carcinogenic, but also important TIME modulators. Their actions may be mediated via multiple mechanisms, including receptor activation, cellular stress responses, epigenetic alterations, chronic inflammation, and impaired immune cell function, which collectively can promote tumor progression and reduce the efficacy of cancer immunotherapies.

We aim to provide a timely overview of how prevalent environmental chemicals, including polycyclic aromatic hydrocarbons, heavy metals, air pollutants, endocrine disruptors, microplastics, and nanoparticles, regulate tumor immunity. It further discusses multiple interconnected aspects, including distinct cellular effects, shared molecular mechanisms, and their impact on the efficacy of cancer immunotherapy. The overarching goal is to provide a theoretical framework for understanding the immune‐related mechanisms underlying environmental carcinogenesis, improving health risk assessment, and exploring translatable strategies for cancer prevention and treatment.

## IMPACT OF PREVALENT ENVIRONMENTAL CHEMICALS ON THE TIME

Polycyclic aromatic hydrocarbons (PAHs) and the dioxin‐like compounds (DLCs), particularly 2,3,7,8‐tetrachlorodibenzo‐p‐dioxin (TCDD), are ubiquitous environmental toxicants generated mainly through incomplete industrial combustion of organic material. Human exposure occurs through inhalation, ingestion, and dermal contact. PAHs can bioaccumulate and are metabolized into more toxic intermediates, whereas TCDD is highly persistent and strongly carcinogenic. Cohort studies have associated environmental PAHs exposure with the progression of multiple human cancers. Within the TIME, these compounds remodel both innate and adaptive immunity.

PAHs and TCDD disrupt TIME homeostasis through distinct mechanisms that ultimately promote tumor progression. At the cellular level, PAHs compromise T cell homeostasis by enhancing regulatory T cell (Treg) differentiation, increasing apoptosis of CD4^+^ and CD8^+^ T cells, and suppressing Th1/Th2 polarization. Meanwhile, in mouse models of hepatocellular carcinoma and lymphoma, TCDD elevates the levels of IL‐1, IL‐2, TNF‐α, TNF‐β, and COX‐2, thereby fostering inflammation‐driven tumor growth [2].

The metalloid Arsenic (As) species exert divergent effects on tumor immunity depending on their chemical form, dose, and experimental context. Inorganic As generally promotes an immunosuppressive tumor microenvironment. It impairs dendritic cell (DC) cytotoxicity and drives M2 macrophage polarization to enhance cancer cells in vitro, while upregulating PD‐1/PD‐L1, expanding Tregs, suppressing pulmonary T cell anti‐tumor function, and promoting lung tumorigenesis in vivo [3]. Interestingly and conversely, arsenic trioxide (ATO) demonstrates potent anti‐tumor immunomodulatory activity. It induces caspase‐3‐dependent Treg apoptosis and reactive oxygen species (ROS)‐dependent immunogenic cell death, inhibits M2 macrophage polarization, and enhances CD8^+^ T cell infiltration in mouse models of colorectal and liver cancers. In addition, ATO can enhance the cytotoxic activity of cytotoxic T lymphocytes in adoptive cell therapy, reduce intratumoral infiltration of Foxp3^+^ Tregs, decrease lung metastasis, and prolong survival in a mouse model of colorectal cancer [4]. Other As species, such as arsenite, similarly potentiate anti‐tumor immunity by downregulating PD‐1, promoting DC maturation and infiltration in acute promyelocytic leukemia mice. These contrasting findings underscore the dual immunological nature of As compounds and highlight ATO's translational potential as an adjuvant in clinical anti‐tumor immunotherapy.

The mechanisms underlying these paradoxical effects likely stem from the divergent redox activity of As species. Unlike elemental As, trivalent As in ATO exhibits substantially stronger oxidizing capacity. ATO functions as a mitochondrial uncoupler of oxidative phosphorylation and avidly reacts with sulfhydryl groups in biological ligands, thereby inhibiting critical matrix enzymes and depleting key intracellular reductants such as cysteine and glutathione. This disruption of redox homeostasis triggers massive ROS generation. Notably, the therapeutic concentration of ATO can be hundreds of folds higher than typical environmental exposure levels, which markedly amplifies oxidative stress. The resulting ROS overload ultimately drives ferroptosis and other forms of immunogenic cell death, reconciling ATO's potent anti‐tumor immunomodulatory effects [5].

Air pollution—including inhalable particulate matter (PM), ground‐level ozone, nitrogen dioxide (NO_2_), and volatile organic compounds (VOCs)—is strongly associated with human cancers, particularly lung cancer [6]. Pollutants such as PAHs and heavy metals adsorbed onto PM synergistically induce oxidative stress and inflammation, disrupt immune homeostasis, and alter the differentiation and function of macrophages, mast cells, T cells, and neutrophils [7]. PM inhalation provokes both pulmonary and systemic inflammation: PM_2.5_ enhances monocyte adhesion to lung adenocarcinoma cells and promotes their proliferation and migration in vitro. Low‐dose PM_2.5_ also amplifies inflammation in acute myeloid leukemia through ROS generation and mitochondrial dysfunction [8]. Diesel exhaust particles rich in PAHs trigger massive neutrophil infiltration and upregulate pro‐inflammatory cytokines (IL‐1β, IL‐6, TNF‐α) and chemokines (CXCL1/2), thereby accelerating lung cancer progression and pulmonary metastasis of breast cancer cells in mice. Of note, although air pollutants generally perturb the TIME to promote tumor progression, certain components can exert paradoxical anti‐tumor effects: NO_2_ enhances macrophage phagocytosis and cytotoxicity, while ozone autohemotherapy promotes Th1 immune responses.

Endocrine‐disrupting bisphenols (BPs) perturb hormone–immune crosstalk by mimicking estrogenic activity via estrogen receptor (ER) signaling. BPA reshapes the TIME toward a pro‐inflammatory yet immunosuppressive state: it shifts the Th1/Th2 balance toward inflammation through MAPK‐dependent chemokine secretion in breast cancer, impairs antigen recognition, and inhibits high‐affinity IgM formation in 4T1 mouse tumors, thereby disrupting humoral immunity [9]. TBBPA, a brominated BPA derivative, independently drives M2 macrophage polarization (CD206^+^/IL‐10^+^/Arg‐1^+^) co‐cultured with breast or endometrial cancer cells [10]. Beyond BPs, other endocrine disruptors such as phthalates similarly suppress M1 and enhance M2 polarization of tumor‐associated macrophages in hepatocellular carcinoma and melanoma, yet mediated via different mechanisms [11].

MNPs enter the human body through ingestion, inhalation, and dermal contact, accumulating in tissues, penetrating biological barriers, and eliciting chronic inflammation [12]. Although whether MNPs can cross the blood–brain barrier remains debated, emerging evidence has confirmed their presence in clinical specimens of breast, pancreatic, gastric, and lung cancers [13]. MNP‐positive tumors exhibit reduced CD8^+^ T cell and NK cell infiltration alongside increased neutrophil accumulation. Meanwhile, MNPs treatment upregulates PD‐L1 expression in gastric cancer cells in vitro, which may confer resistance of anti‐tumor immunity [14]. Like PM, MNPs also function as vectors for environmental chemicals such as PAHs and heavy metals, enhancing their mobility and producing synergistic effects that reshape the TIME and amplify immunotoxicity [15].

In addition to traditional chemical pollutants, the interactions between emerging environmental factors and the TIME are beginning to attract attention. Among them, per‐ and polyfluoroalkyl substances (PFAS), known as “forever chemicals,” have become a major research focus due to their environmental persistence and widespread distribution. In an ovarian cancer mouse model, PFAS exposure promotes immunosuppression via Wnt/β‐catenin‐dependent Treg infiltration and M2 macrophage polarization. Bioinformatic analyses of public database suggest that PFOA may regulate the expression of immune‐related genes such as *CXCL11* and *PDE2A* [16]. However, despite extensive research on PFAS–immune system associations and PFAS–cancer links independently, studies that explicitly bridge these two domains by directly examining the effect of PFAS on the TIME remain scarce.

A critical yet underexplored question is whether environmental exposures compromise cancer immunotherapy efficacy. The converging effects of environmental chemicals, such as Treg expansion, cytotoxic CD8^+^ T cell exhaustion, and M2‐type tumor‐associated macrophage polarization reviewed here, offer a plausible basis for such interference. Collectively, these alterations forge a suppressive TIME that possibly undermine immune checkpoint inhibitors. The resultant T cell dysfunction may also extend to adoptive cell therapies and cancer vaccines: CAR‐T cells and vaccine‐induced effector T cells require functional competence for both priming and cytotoxic execution, and the T cell exhaustion state driven by environmental chemical exposures likely impairs both phases.

## MOLECULAR MECHANISMS UNDERLYING TIME REMODELING BY ENVIRONMENTAL CHEMICALS

Receptor activation represents a primary mechanism by which environmental chemicals modulate the TIME. Among these, the aryl hydrocarbon receptor (AhR) serves as a key sensor for environmental stimuli and is frequently activated during cancer progression. PAHs and DLCs directly engage AhR, which triggers multiple immunosuppressive programs. Activated AhR upregulates inflammatory and chemotactic mediators, including IDO2, IL‐4, IL‐6, CXCR2, CXCR4, and ARG‐1. Concurrently, it upregulates BCL‐6 expression, which suppresses CD80 and CD69 levels in B cells. This dysregulation impairs humoral immunity and promotes the development of non‐Hodgkin lymphoma [2]. In parallel, the ER, particularly ERα, represents another pathway potentially linking environmental exposure to TIME remodeling [17]. ERα activation directly downregulates eosinophil peroxidase expression and attenuates its cytotoxicity within the breast cancer microenvironment [18]. Nevertheless, although both endocrine‐disrupting chemicals and ER activation have been independently shown to influence the TIME, direct evidence showing these chemicals mediate their immunomodulatory effects specifically through ER signaling remains limited, which warrants thorough loss/gain of function validations.

Cellular stress responses have also been frequently implicated in linking environmental chemical exposure to TIME remodeling. Mitochondrial dysfunction and oxidative stress represent one major axis: PM_2.5_ elevates ROS and NADPH oxidase to drive proliferation of leukemic cells, while MNPs penetrate skin cancer cells, increase mitochondrial ROS, and activate the NLRP3 inflammasome to promote tumor growth [12]. Intriguingly, ROS can induce entirely different immunological outcomes under varying conditions, while its effect appears to be determined not only by its abundance but also by the cellular source, mode of generation, and the microenvironment. Meanwhile, endoplasmic reticulum stress response, which has been extensively implicated in both cancer and immune cells within the TIME [19], represents another parallel mechanism. Our recent work demonstrated that phthalates trigger the IRE1α‐XBP1s axis of the unfolded protein response in liver cancer, fostering M2 macrophage polarization through cholesterol metabolic reprogramming [11]. These observations underscore the challenge and potential of therapeutically targeting cellular stress responses in TIME disturbance induced by environmental chemicals.

Importantly, the pathways by which environmental factors reshape the TIME are interconnected rather than operating in isolation. For example, AhR activation promotes ROS generation and c‐Src activation in tumor cells; activated c‐Src subsequently stimulates epidermal growth factor receptor signaling and downstream kinase pathways. Dysregulation of these pathways in turn induces mitochondrial dysfunction, altered membrane potential, and perturbed calcium homeostasis, ultimately triggering endoplasmic reticulum and mitochondrial stress responses. Finally, depending on the specific combination and dosage of environmental chemicals, as well as host species and sex differences, their biological effects may vary substantially and can even be opposing under different conditions [20].

## CONCLUSION AND PROSPECTS

**Figure 1:**
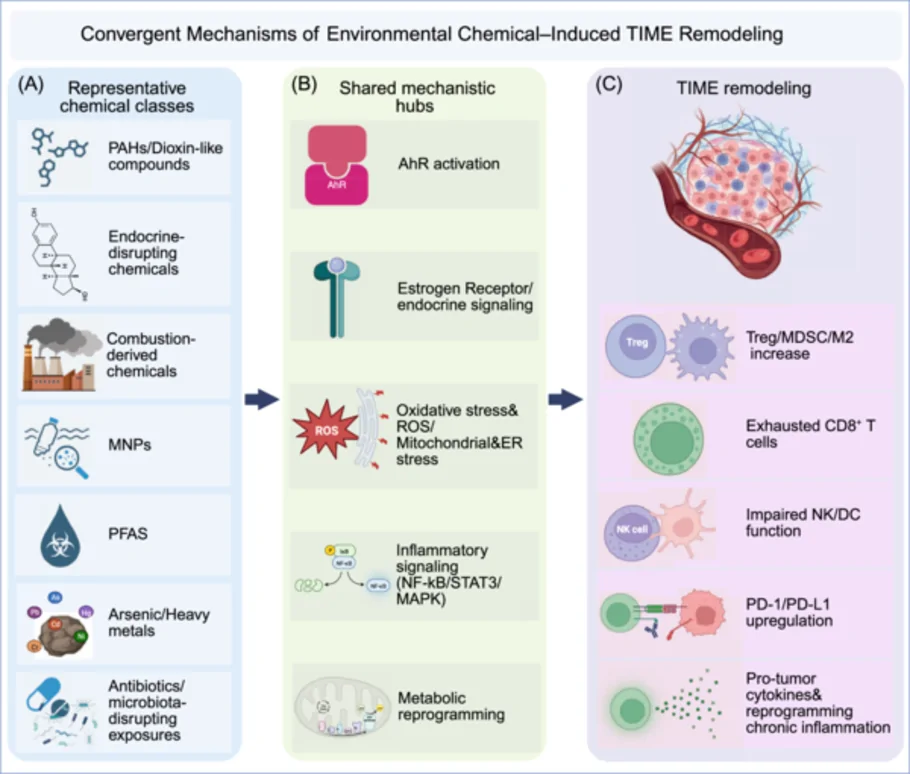
Convergent mechanisms of environmental chemical‐induced time remodeling. (A) Various environmental chemicals, including polycyclic aromatic hydrocarbons (PAHs), heavy metals, air pollutants, endocrine disruptors, and microplastics and nanoparticles (MNPs), (B) collectively regulate tumor immune homeostasis through a complex network of mechanisms, encompassing specific receptor activation, organelle dysfunction, cellular stress response, metabolic reprogramming, as well as the activation of inflammation‐related signaling pathways. (C) These diverse pathways ultimately converge on the tumor immune microenvironment (TIME), altering its immunometabolism, upregulating immune checkpoint expression, and modulating the composition and function of tumor‐infiltrating immune cells, thereby impacting antitumor immunity. AhR, aryl hydrocarbon receptor; DC, dendritic cell; ER stress, endoplasmic reticulum stress; MDSC, myeloid‐derived suppressor cell; NK, natural killer cell; PFAS, per‐ and polyfluoroalkyl substances; ROS, Reactive oxygen species; Treg, regulatory T cell.

To sum up, we summarize the effects and molecular mechanisms of prevalent environmental chemicals on tumor immunity (Figure 1A–C). Current studies suggest that environmental chemical exposure commonly promotes M2 macrophage polarization, Treg expansion, and CD8^+^ T cell exhaustion, while potentially reducing the efficacy of cancer immunotherapies.

**Figure 2:**
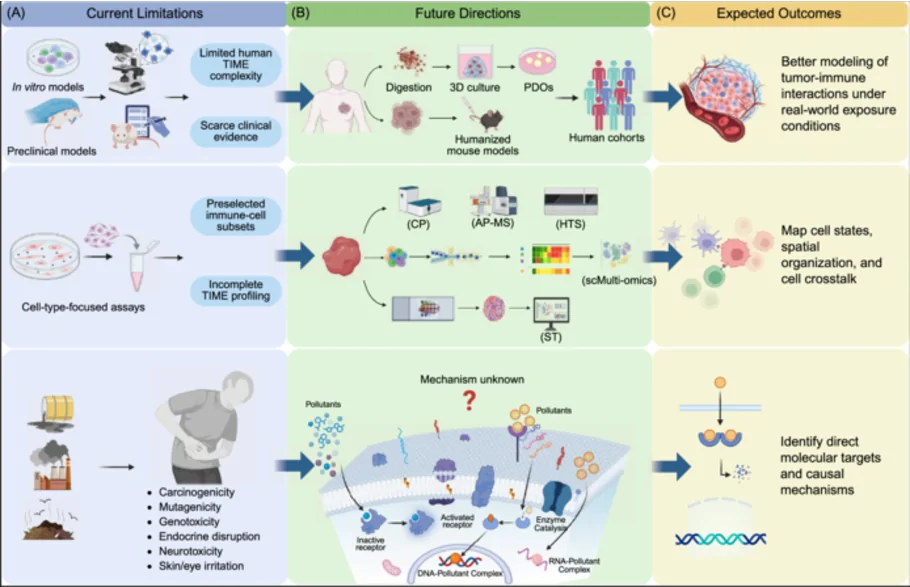
Current limitations and future perspectives. Despite the increasing number of ongoing studies, current research still faces limitations and challenges. (A) This includes a lack of clinical and human‐level evidence, insufficient dissection of molecular initiating events, inadequate utilization of advanced technologies, and difficulty in simulating real‐world environmental chemical exposure conditions. (B) Future studies could be improved by adopting more representative experimental models, employing advanced technological approaches, and evaluating the combined exposure to multiple environmental factors. (C) This will lead to findings that more closely reflect human exposure scenarios, deepen the understanding of how environmental chemicals act on and regulate the TIME, and lay a solid molecular foundation for effective prevention and intervention strategies. AP‐MS Affinity Purification – Mass Spectrometry; CP Chemical Proteomics; HTS High‐Throughput Sequencing; PDOs, Patient‐Derived Organoids; ST Spatial Transcriptomics.

Despite these advances, research in this field remains at an early stage and faces several important limitations (Figure 2A). First, most studies rely on in vitro or preclinical models that do not fully recapitulate the three‐dimensional architecture, dynamic cellular interactions, and stromal complexity of the human TIME. Epidemiological and clinical data reflecting real‐world scenarios remain scarce, limiting the translational relevance of current findings. Second, despite compositional and functional alterations within the TIME, the initial molecular interactions triggered by environmental chemicals remain poorly dissected. In particular, interactions with cell surface receptors, intracellular proteins, nucleic acids, and other biomolecules may represent critical early events that determine downstream immunological outcomes. Third, although single‐cell sequencing and spatial transcriptomics now allow high‐resolution profiling of the TIME, their application in environmental toxicology remains limited, and the precise immune cell targets and spatiotemporal effects of chemical exposure therefore remain unclear. Fourth, environmental chemicals typically exist as complex mixtures in real‐world settings, whereas most current studies focus on single agent or simple combinations, leaving the synergistic, additive, or antagonistic effects of realistic multi‐component exposures poorly understood. Finally, how variables such as chemical dose, exposure duration, and individual (epi)genetic susceptibility modulate these immunological effects remains poorly understood.

To address these challenges, we propose the following priorities of future research (Figure 2B,C): (i) advance model fidelity by deploying patient‐derived materials, humanized mice, and multicellular 3D systems that preserve stromal–immune crosstalk, coupled with prospective clinical cohorts to strengthen exposure–outcome associations; (ii) map molecular initiating events via biochemical approaches to identify the full spectrum of proteins, nucleic acids, and receptors engaged by environmental chemicals at the onset of action; (iii) resolve cellular vulnerability and spatial context by integrating single‐cell multi‐omics with spatial transcriptomics to pinpoint susceptible immune subsets, trace their altered developmental trajectories, and localize these interactions within the TIME; (iv) embrace real‐world complexity by constructing representative mixture models and leveraging exposomics to dissect the synergistic or antagonistic immunomodulatory effects of complex chemical mixtures. Prospective studies in these directions will deepen our understanding of how environmental chemicals act upon and modulate the TIME, laying a solid molecular foundation for effective preventive and interventional strategies.

## AUTHOR CONTRIBUTIONS

**Tongtong Zhang**: Writing—original draft; writing—review and editing. **Yanxia Hu**: writing—review and editing; supervision. **Weiran Zhang**: Visualization; writing—original draft; writing—review and editing. **Yang Jin**: Project administration; supervision; writing—review and editing. **Chaoyang Sun**: Writing—review and editing; supervision; resources. **Afshin Samali**: Writing—review and editing; writing—original draft; supervision. **Shunqing Xu**: Writing—review and editing; supervision; resources. **Xia Sheng**: Writing—review and editing; conceptualization; supervision; resources; project administration; funding acquisition.

## CONFLICT OF INTEREST STATEMENT

The authors declare no conflicts of interest.

## ETHICS STATEMENT

No animals or humans were involved in this study.

## AI STATEMENT

DeepSeek‐R1 was used for language editing. All content has been verified for accuracy by the authors.

## Data Availability

Data sharing is not applicable to this article as no datasets were generated or analyzed during the current study. No new data and scripts were generated in this Commentary. Supplementary information (graphical abstract, slides, videos, Chinese translated version, and updated materials) may be found in the online DOI or iMeta Science https://www.imeta.science/.
